# Chikungunya seroprevalence in the Horn of Africa: A systematic review and meta-analysis

**DOI:** 10.1097/MD.0000000000047564

**Published:** 2026-02-06

**Authors:** Yishak Abraham, Dawit Getachew Assefa, Kibrom Abraham, Monica S. Kahabuka, Esther Nthenya Muthoka, Seke G.Y. Muzazu, Mesoud A. Bushara, Firehiwot Ayenadis, Tsegahun Manyazewal

**Affiliations:** a Addis Ababa University, College of Health Sciences, Center for Innovative Drug Development and Therapeutic Trials for Africa (CDT-Africa), Addis Ababa, Ethiopia; b Department of Nursing, College of Health Science and Medicine, Dilla University, Dilla, Ethiopia; c John Snow, Inc. (JSI), Addis Ababa, Ethiopia; d Kibong’oto National Infectious Diseases Hospital, Kilimanjaro, Tanzania; e Tororo General Hospital, Tororo, Uganda; f Enteric Diseases and Vaccines Research Unit, Centre for Infectious Disease Research in Zambia (CIDRZ), Lusaka, Zambia; g Faculty of Geographical and Environmental Sciences, University of Khartoum, Khartoum, Sudan; h Addis Ababa Burn, Emergency, and Trauma Hospital (AaBET Hospital), St. Paul’s Hospital Mellinium Medical Collage, Addis Ababa, Ethiopia.

**Keywords:** chikungunya virus, Djibouti, Eritrea, Ethiopia, Horn of Africa, Kenya, Somalia, South Sudan, Sudan, Uganda

## Abstract

**Background:**

Chikungunya virus (CHIKV) poses a significant burden on affected populations, presenting substantial challenges to public health. This study aimed to assess the seroprevalence of the CHIKV in the Horn of Africa.

**Methods:**

We conducted a systematic review and meta-analysis by searching PubMed/MEDLINE, Scopus, Scientific Direct, Google Scholar, and reference lists for primary articles published from the inception of the database until November 30, 2023. The inclusion criteria covered seroprevalence studies of CHIKV in Ethiopia, Kenya, Somalia, South Sudan, Sudan, Eritrea, Uganda, and Djibouti. Pooled seroprevalence was estimated using a random effects model, and the meta-analysis was conducted with R Studio version 4.3.1 and the Metapro package. The study protocol adhered to the Preferred Reporting Items for Systematic Reviews and Meta-Analyses guidelines and is registered in PROSPERO, CRD42023477057.

**Results:**

From a pool of 87,567 potential studies, 34 eligible studies were included in our analysis. Most of the studies were conducted in Kenya (44%). Hospital-based studies were included in 59% of cases. A total of 23,400 participants were involved in the review. Of the 13,397 participants, 6778 (67.6% of those with information) were male. The pooled seroprevalence of CHIKV was 14% (95% CI: 9–23; *I*^2^ = 99%). Subgroup analysis was performed. The seroprevalence was higher in studies conducted in population settings: 15% (95% CI: 5–37; *I*^2^ = 99%) than in hospital settings. The seroprevalence of chikungunya was high from the 2004 to 2013 period, at 36% (95% CI: 13–68; *I*^2^ = 98%). Plaque reduction neutralization tests detected 15% (95% CI: 3–49%; *I*^2^ = 94%) of the chikungunya seroprevalence. The seroprevalence of CHIKV among inapparent infections was 17% (95% CI: 8–35; *I*^2^ = 98%). The meta-regression analysis revealed that the chikungunya seroprevalence was predicted by the countries of study, age group, and trends of infection over time.

**Conclusion:**

Our review highlights compelling evidence of CHIKV and other arbovirus circulation in the Horn of Africa, revealing diverse seroprevalence rates across different countries, age groups, laboratory tests, clinical manifestations, and time trends. The confirmatory gold standard, the plaque reduction neutralization test, increases diagnostic accuracy.

## 1. Introduction

Chikungunya is a mosquito-borne viral disease caused by chikungunya virus (CHIKV), a RNA virus in the alphavirus genus of the family Togaviridae.^[1]^ It is transmitted to humans through the bite of female *Aedes* spp. mosquitoes (mostly *Aedes aegypti* and *Aedes albopictus*). The virus was identified in 1952 in the Newala district of Tanzania, where it was named from the language of the Makonde people, meaning “bends up,” pointing to severe joint pain.^[2]^ Since then, the virus has evolved into 4 major genotypes, the East-Central-South-African and West African genotypes, which are associated with epidemics in the sub-Saharan regions, and the Asian genotype and the Indian Ocean lineage, which are linked with epidemics in parts of Asia and the Indian Ocean islands.^[3]^

Chikungunya symptoms include fever, headache, rash, fatigue, myalgia, and severe polyarthralgia.^[4]^ The incubation period ranged from 1 to 12 days.^[5]^ While the acute symptoms generally last from a few days to 2 weeks, the debilitating polyarthralgia can persist for months or even years in a significant proportion of patients, leading to chronic morbidity.^[6]^ Notably, certain individuals, particularly those of advanced age or with comorbid conditions, may experience an atypical presentation of the disease.^[6,7]^ This atypical form can include severe neurological, dermal, or cardiovascular complications, and even mortality, diverging from the classic symptomatology. The clinical presentation of CHIKV infection shares significant overlap with other arboviruses. Dengue fever is more common in the tropics and subtropics. It commonly presents with a high fever, headache, body aches, nausea, and rash.^[8,9]^ Most patients will also improve within 1 to 2 weeks and have an asymptomatic presentation. Zika virus generally has mild symptoms, including rash, fever, conjunctivitis, muscle and joint pain, malaise, and headache, and usually lasts for 2 to 7 days.^[10]^ Another common arbovirus is yellow fever. Symptoms include fever, headache, muscle pain, nausea, vomiting, or loss of appetite.^[11,12]^ In most cases, symptoms disappear after 3 to 4 days. O’nyong-nyong virus (ONNV) is clinically associated with fever, polyarthralgia, rash, and lymph node involvement.^[13]^ Unlike other arboviruses such as chikungunya, dengue fever, Zika, and yellow fever, it is transmitted by *Anopheles* mosquitoes and tends to occur in areas where malaria is also common.^[14]^ It is genetically and antigenically closely related to CHIKV.^[15,16]^ Adding to the diagnostic challenge is the possibility of co-infections with multiple arboviruses circulating in the same region. These clinical similarities and the potential for co-infections make diagnosis very difficult.

The diagnostic modalities used for chikungunya are serologic and molecular tests.^[17]^ Serologic testing includes viral antigen–antibody detection, which is achieved by using an immunoglobulin M (IgM)/immunoglobulin G (IgG) enzyme-linked immunosorbent assay (ELISA), an immunofluorescence assay (IFA), hemagglutination inhibition test (HI), a rapid diagnostic test, and immunoblotting methods. They are used in the acute phase and have different sensitivities and specificities. In addition, these tests are easier to perform, cost-effective, and require minimal resources than molecular tests.^[18]^ However, because of the cross-reactivity of recombinant CHIKV E1 and E2-encoded proteins with the ONNV, confirmatory tests such as plaque reduction neutralization tests (PRNTs) are needed.^[19]^ Moreover, molecular techniques such as reverse transcription-polymerase chain reaction (RT-PCR) and isothermal amplification remain the gold standards for diagnostic tests.^[20]^

Several factors facilitate the spread of CHIKV such as population density, human migration, urbanization, lack of immunity, and vector adaptations, inadequate vector control programs further contribute to outbreak potential.^[21–23]^ In addition, studies have shown that the incidence of chikungunya varies with factors such as occupation, age, sex, education, and outdoor activities.^[24]^ Farmers and older people are associated with higher prevalence rates.^[25]^

CHIKV outbreaks have been reported globally, with a significant burden in Africa. The continent has experienced numerous epidemics, and in the context of increasing globalization and human migration, these outbreaks have frequently spread beyond their initial foci.^[26,27]^ The 1st case of CHIKV in Ethiopia was identified in the Dollo Ado district, Somali region of Ethiopia, in June 2016, and it was imported from the border of Mandera County, Kenya.^[28]^ Subsequently, outbreak cases were reported in the Dire Dawa city administration, the Ad’ar district of the Afar region, the Somali region, and the Dawro zone.^[29–32]^ In 2004, Kenya experienced an outbreak.^[33]^ Sudan^[34–36]^ and Djibouti^[37]^ have also experienced outbreaks.

The potential for disease outbreaks, co-infections with other arboviruses, severe and atypical forms, as well as the lack of vaccines and difficulty in controlling the disease, highlight the importance of scientific knowledge regarding the true burden of chikungunya. As a result, the objective of this systematic review and meta-analysis was to assess CHIKV seroprevalence and highlight its coinfections with other arboviruses in the Horn of Africa. The aim of measuring CHIKV seroprevalence in the region is to help public health officials understand virus propagation, identify high-risk locations, and improve disease control and prevention initiatives between nations across borders.

## 2. Methods

A systematic review and meta-analysis were conducted, and the report followed the Preferred Reporting Items for Systematic Reviews and Meta-Analyses (PRISMA) guidelines.^[38]^ The protocol was prospectively registered with PROSPERO, CRD42023477057.

### 2.1. Eligibility criteria

The condition, context, and population (CoCoPop) description model was used to set eligibility criteria for the study.

Condition: CHIKV seroprevalence in populations is determined by laboratory diagnosis of prior or current infection by antibody detection methods such as ELISA using IgG and IgM, IFA, and HI. Studies that included outbreaks and used RT-PCR tests only were excluded from the review.

Context: reported seroprevalence studies of CHIKV in the Horn of Africa which includes Ethiopia, Kenya, Somalia, South Sudan, Sudan, Eritrea, Djibouti, and Uganda, were included.

Populations: the review covered all studies done across all age groups.

Study design: observational studies, such as case–control, cross-sectional, and cohort studies, were included in the review.

Review articles, editorials, commentaries, conference abstracts and studies for which the full text is not available were excluded. In addition to this studies not published in English language were excluded.

### 2.2. Search strategy

A systematic literature search was performed to identify relevant articles from the online databases. PubMed/MEDLINE, Scopus, Science Direct, and Google Scholar, and other reference lists were searched for primary articles published from the inception of the database until November 30, 2023. The search was performed in accordance with the recommendations in the Cochrane Handbook for Systematic Reviews of Interventions.^[39]^

The search strategies used in PubMed for the text words were as follows: (((((“chikungunya virus”[MeSH Terms] OR (“chikungunya”[All Fields] AND “virus”[All Fields]) OR “chikungunya virus”[All Fields] OR (“chikungunya fever”[MeSH Terms] OR (“chikungunya”[All Fields] AND “fever”[All Fields]) OR “chikungunya fever”[All Fields])) AND (“seroepidemiologic studies”[MeSH Terms] OR (“seroepidemiologic”[All Fields] AND “studies”[All Fields]) OR “seroepidemiologic studies”[All Fields] OR “seroprevalence”[All Fields] OR “seroprevalences”[All Fields] OR “seroprevalance”[All Fields] OR “seroprevalances”[All Fields] OR “seroprevalency”[All Fields] OR “seroprevalent”[All Fields])) OR “Seroepidemiology”[All Fields]) AND (“ethiopia”[MeSH Terms] OR “ethiopia”[All Fields] OR “ethiopia s”[All Fields])) OR (“somalia”[MeSH Terms] OR “somalia”[All Fields] OR “somalia s”[All Fields]) OR (“sudan”[MeSH Terms] OR “sudan”[All Fields] OR “sudans”[All Fields] OR “sudan s”[All Fields]) OR (“south sudan”[MeSH Terms] OR (“south”[All Fields] AND “sudan”[All Fields]) OR “south sudan”[All Fields]) OR (“kenya”[MeSH Terms] OR “kenya”[All Fields] OR “kenya s”[All Fields]) OR (“eritrea”[MeSH Terms] OR “eritrea”[All Fields]) OR (“djibouti”[MeSH Terms] OR “djibouti”[All Fields]) OR (“uganda”[MeSH Terms] OR “uganda”[All Fields] OR “uganda s”[All Fields])).

### 2.3. Study selection

We used the Cochrane Handbook for Systematic Reviews of Interventions^[39]^ and R Studio version 4.3.1 Metapro package for data management and analysis. Two authors (YA and DGA) independently reviewed the results, and disagreements were resolved through discussion.

### 2.4. Data extraction and management

The title and abstract were retrieved from the electronic search and reference lists and independently screened by 2 authors. Data were extracted from each included study using a standardized form, including information on the author, journal and year of publication, country and city, period, study design, study population, age, sex, laboratory test, clinical symptoms, number of positive results (event), and total sample size.

### 2.5. Risk of bias assessment

The Agency for Healthcare Research and Quality checklist for a cross-sectional study design was used to assess the risk of bias (ROB).^[40,41]^ If the quality of the study fulfilled the methodological requirement, 1 score was assigned to each item. A score of 0 to 4 indicates a high ROB, 5 to 7 indicates a moderate ROB, and 8 to 11 indicates a low ROB. Two reviewers (YA and MSK) performed the assessments separately, and discrepancies were resolved through discussions with a third reviewer (TM).

### 2.6. Data synthesis

The pooled seroprevalence of CHIKV in the study population was calculated using meta-analysis methods, including a random effects model, to account for heterogeneity between studies.

### 2.7. Assessment of heterogeneity

Forest plots were constructed to assess heterogeneity among the included studies. The Cochrane *Q* and *I*^2^ statistics, which measure the percentage of variance resulting from true differences in the effect sizes rather than the sampling error, were used to measure heterogeneity among the studies.^[42]^ We performed subgroup analyses according to the key potential sources of heterogeneity among the countries of study, study settings, laboratory diagnostic tests used, 3 age groups, trends of infection over time and clinical manifestations. Furthermore, given the high degree of heterogeneity of the true differences in the effect sizes, we ran a meta-regression to regress the prevalence. A funnel plot and Egger regression test were used to assess publication bias in the studies.^[43]^ To determine statistical significance, the Chi^2^ test with a *P* value of <.05 was used.

## 3. Results

**Figure 1.**
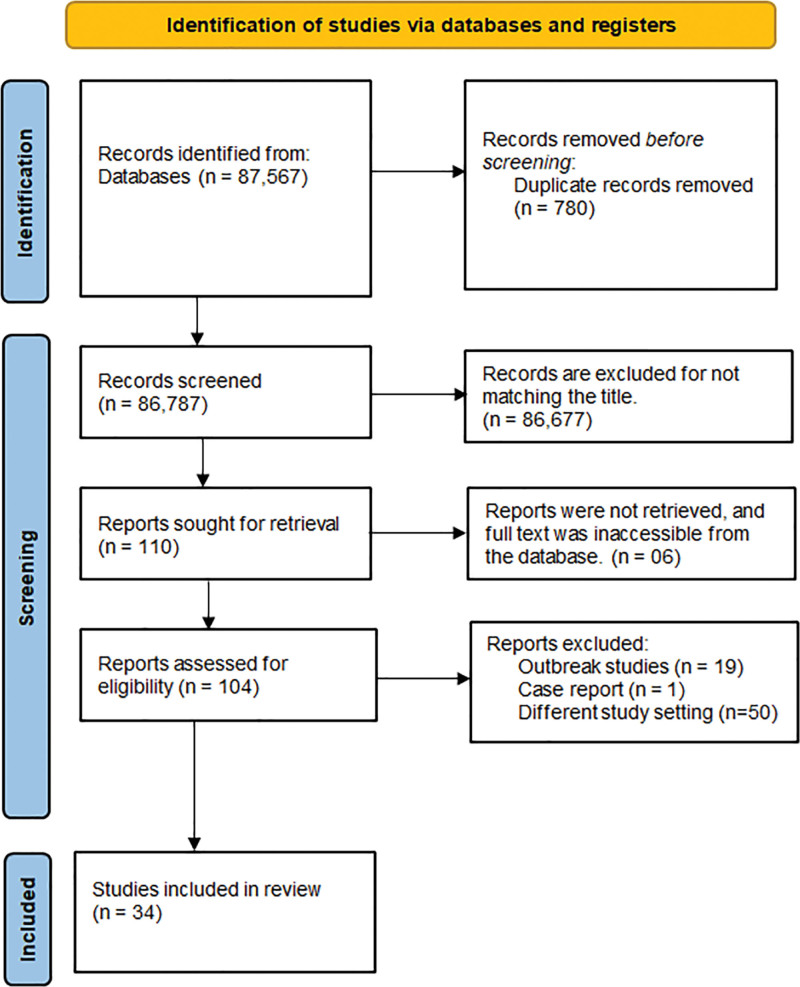
PRISMA flow diagram illustrating the study selection process for the systematic review and meta-analysis of chikungunya virus seroprevalence in the Horn of Africa. PRISMA = Preferred Reporting Items for Systematic Reviews and Meta-Analyses.

The search resulted in a total of 87,567 studies, of which 54 full-text eligible studies were evaluated further; 34^[33,44–76]^ of these studies fulfilled the inclusion criteria and were included in the meta-analysis and qualitative analysis (Fig. 1).

### 3.1. Study characteristics

Total of 23,400 participant involved in the review. Most of the studies were conducted in Kenya (44%),^[33,54,55,57–62,64,67,68,73–75]^ Sudan (23.5%),^[45,46,52,65,67,69,72,76]^ Uganda (14.7%),^[49,50,56,63,70]^ and Ethiopia (8.8%).^[47,51,53]^ Fifty-nine percent of the studies were hospital-based,^[33,45,46,49–51,53,56–58,60,62,63,65–67,69,70,75,76]^ and 38% of the studies were population based.^[33,44,47,48,52,54,55,59,61,64,68,73,74]^ Of the 13,397 participants, 6778 (67.6% of those with information) were male. Seventy-three percent of the studies were performed among all age groups, while 15% and 12% of the studies were performed among children (<18 years) and adults (>18 years), respectively. Out of 34 studies, 43% were conducted in participants with inapparent infections^[44,47,50–52,54,57,61,64,66,68,70–73]^ and 43% in symptomatic infections,^[44,46,47,51,56–58,60,61,63,65,69,74,75]^ whereas 14% of studies^[33,55,56,69,74]^ did not clearly state the clinical symptoms information in the primary studies. ELISA was only performed for 65% of the studies. Of these, 54.5% were IgG and 41% were IgM/IgG. Only the hemagglutination test was performed for 23.5% of the studies. Moreover, a PRNT was performed for 44% of the studies. Among those coinfected with arboviruses, 32% were infected with dengue virus,^[44,45,49,50,55,57,59,64,68,70,73]^ 38% with yellow fever,^[44,47–51,54,56,57,64,70,72,73]^ 11.7% with Zika virus,^[47–49,54]^ 11.7% with Rift Valley virus,^[44,48,64,68]^ and 17.6% with West Nile virus.^[48–50,54,57,72]^ Malaria coinfection occurred in 1 study.^[59]^ The review revealed that the common reasons for the high chikungunya seroprevalence in the Horn of Africa were household density, large family size, sleeping in open at night, travel history to forest, favorable breeding sites for vectors, climate and environmental fluctuations, farming/irrigation, long rainy seasons and frequent outbreaks in the region.^[44,47,51,53–55,57–59,74]^ The study design, demographic information, and other information are summarized in Table 1 and diagnostic laboratory tests performed were summarized in Table S1, Supplemental Digital Content, https://links.lww.com/MD/R324.

**Table 1:** Summary characteristics of the studies included in the systematic review and meta-analysis.

No.	Reference	Study design	Study setting	Country	Sex (N), positive cases (N)		Age group (yr)	Lab test	Clinical presentation	Event	Study population	Additional test	Reasons for increased seroprevalence of CHIKV
					M	F							
1	Adam^[45]^	Cross-sectional study	Hospital based	Sudan	166 (–)	213 (–) ,	<15 = 42, 15–30 = 119, 31–45 = 116, >45 = 102	IgG (ELISA)	Acute febrile illness symptom	7	379	All ELISA + were also IFA + and VNT+. 7/379 were also DENV +.	Mosquitoes in Sudan have low vector competence for CHIKV.
2	Alafra A^[46]^	Cross-sectional study	Hospital based	Khartoum State, Sudan	47 (1)	43 (2)	<20 = 18 (1) 21–30 = 38 (2) 31–50 = 32 (0) 51–70 = 2 (0)	IgM (ELISA)	Arthritis patients	3	90	6 were CHIKV qRT-PCR+	–
3	Andayi^[44]^	Cross-sectional study	Population based	Djibouti	466 (12)	571 (12)	≤19 = 409 (8) 20–39 = 435 (13) 40–59 = 155 (1) ≥60 = 36 (2)	IgG (ELISA)	No symptom	24	914	96% (23/24) of ELISA + were VNT+. DENV+ were 199/911 (21.8%), WNV + were 5/893 (0.6%), YFV + was 14/903 (1.5%) and RVF + was 20/914 (2.2%).	Large family size in the household (>6) [AOR 5.7 95% CI 2.6–12.5], sleeping in open at night [AOR 3.4 95% CI 1.3–8.4] and densely populated city center [AOR 8.2 95% CI 2.8–23.9] was risk factor for high CHIKV prevalence.
4	Asebe^[47]^	Cross-sectional study	Population based	Gambella region, Ethiopia	53 (12)	37 (2)	18–30 = 36 (4) 31–40 = 25 (4) ≥41 = 29 (6)	IgG (ELISA)	No symptom. No YF vaccination history.	14	90	2.9% (4/135) were IgG + for YFV; 27.3% (41/150) were IgG + for ZIKV.	Community border with south Sudan and Kenya, most are agro- pastoralist with travel history to forest area.
5	Botros^[48]^	Survey	Population based (refugee camp)	Somalia	–	–	–	HI	Some had fever	0	28	0% (0/10) of convalescent sample tested were HI +; 0% (0/28) were HI + for SNIV,WNV,YFV and RVF.	-
6	Byaruhanga^[49]^	Cohort	Hospital based	Uganda	732 (–)	709 (–)	0–14 = 317 15–34 = 621 35–54 = 346 55–64 = 84 >65 = 73	IgM (ELISA)	Febrile illness patients	58	1441	137 were PRNT + for alphavirus, out of 3.6% (5/137) were CHIKV+, 13 (18/137) were ONNV+. YFV (34/1441), WNV (19/1441), DENV (14/1441) and ZKV (13/1441).	–
7	Clements^[50]^	Survey	Hospital based	Fort Portal, Mbarara Kampala, Mabale and Gulu, Uganda	–	–	–	IgG (ELISA)	Healthy blood donors	552	1744	4.4% (4/552) of CHIKV IgG-positive samples were PRNT+. Other virus also co-exist in the blood sample like YFV, DENV2, and WNV; antibody prevalence was 3.3% (58/1744), 4.1% (48/1744), and 8.3% (144/1744), respectively. Sampled test showed PRNT + cross-reaction with ONNV (23 samples).	–
8	Endale^[51]^	Cross-sectional study	Hospital based	South Omo Valley, Ethiopia	186 (74)	174 (83)	5–10 = 17 11–20 = 62 21–35 = 173 36–55 = 86 >55 = 22	IgG (ELISA)	No symptom	157	360	49.5% (155/313) were IgG + for YFV. Cross-reactivity test was not done for ONNV and other alphavirus.	The presence of favorable breeding site, area was endemic to YFV (reservoirs for CHIKV and for many other arboviruses).and the proximity of the present study area to Kenya.
9	Farnon^[52]^	Survey	Population based	Kortalla, Sudan	37 (–)	50 (–)	Median age = 32yr	IgM (ELISA)	No symptom	37	87	1% (1/87) was CHIKV IgM+; 7.9% (3/38) of CHIKV + sample were SNIV+.	
10	Ferede^[53]^	Cross-sectional study	Hospital based	Northwest, Ethiopia	388 (104)	198 (28)	≤14 = 116 15–29 = 296 30–44 = 112 ≥64	IgM/IgG (ELISA)	Fever	135	586	22.5% (132/135), 5.3% (31/135), and 4.8% (28/135) were IgM+, IgG+, and IgM and IgG+, respectively.	Risk factors includes being male (outdoor activity) and presence of stagnant water.
11	Geser^[54]^	Survey	Population based	Central Nyanza, Kitui District and Malindi District, Kenya	–	–	0–19 = 1103 20–39 = 834 40–59 = 522 >60 = 231 Unknown = 8	HI.	No symptom	883	2698	SBV 12.2% (330/2698); ZKV 17.6% (475/2698); YFV 14.3% (387/2698) and WNV 26.5% (715/2698)	High density of houses in the districts associated with high arbovirus infection. There is no association with distance from insect breeding grounds or the size of the household.
12	Grossi-Soyster^[55]^	Cross-sectional study	Population based	Town of Busia, western Kenya	239 (–)	260 (–)	5–14 = 250 15–34 = 138 35–54 = 79 55–74 = 40 >75 = 2	IgG (ELISA)	–	335	499	No other test was done. (8/500) were DENV +	Climate and environment fluctuate between drought and flooding, individuals herding and grazing livestock may have an increased risk.
13	Henderson^[56]^	Survey	Hospital based	Kigezi, Karamoja, Mengo A and B, West Nile A and B, Madi district, Bwamba, Uganda	- (–)	- (–)	<15 = 828 >15 = 1041	HI	–	722	1869	12.6% (236/1869) were HI + for SNV; 3.7% (69/1869) were HI + for YFV	-
14	Inziani^[57]^	Cross-sectional study	Hospital based	Teso South Sub-County/Busia County, Kenya	316 (–)	340 (–)	<12Yr	IgG/IgM	No symptom	36	649	42/54 (77.8%) were PRNT + for CHIKV; 1.4% (5/368) of DENV1+; 9.0% (50/656) of DENV2+; 19.7% (40/203) of DENV3+; 9.6% (62/649) of WNV+; 4.4% (29/656) of YFV+	Due to their immature immune system.
15	Kamau^[58]^	Cross-sectional study	Hospital based(Laboratory sample)	Western Kenya	145 (–)	237 (–)	Mean age = 32	IgM/IgG (ELISA)	Acute febrile illness symptoms	114	382	27% (29/107) and 4.8% (5/107) had CHIKV and ONNV specific neutralizing antibodies respectively	Long rains with peak transmission in July, travel history to forest (collect woods) and open water sources.
16	Khan^[59]^	Cohort	Population based	Western and costal Kenya	1688 (–)	1757 (–)	7 (5–10 IQR)	IgG (ELISA)	Acute febrile illness symptoms	320	3444	5.3% (181/3443) were DENV+, 19.7% (2037/10,362) were malaria+.	Household crowding (HCI > 2), presence of litter, and high wealth index regarded as risk factor for increased cases.
17	Kimata^[60]^	Retrospective study	Hospital based (laboratory)	Nairobi County, Coastal region and North-Eastern region,Kenya	234 (–)	158 (–)	4.7 (2.8–6.7 IQR)	IgM/IgG (ELISA)	Measles like symptoms	18	392	11% (2/18), 39% (7/18) and 50% (9/18) had neutralizing antibodies for CHIKV, ONNV, and both CHIKV and ONNV. No positive SNIV and SFV.	-
18	LaBeaud^[61]^	–	Population based	Coastal Kenya	–	–	Mean age 21.7 yr (7–54yr)	IgG (ELISA)	No symptom	486	1848	443 were PRNT+, out of which 6% (25/443) were CHIKV+, 56% (250/443) were ONNV+, and 38% (168/443) were CHIKV and ONNV+	–
19	Masika^[62]^	Cross-sectional study	Hospital based	Rural (Taita-Taveta) and urban (Kibera, Nairobi) in Kenya	261	286	Mean age = 22..4 (2month- 85 yr)	IgG(IFA) IgM (ELISA)	Febrile patients	86	557	1.4% (1/69) were PRNT + for CHIKV; 20% (14/69) were PRNT + for CHIKV, and ONNV and 21.7% (15/69) were PRNT + for ONNV only.	–
20	McCrae^[63]^	Survey	Hospital based	Entebbe, Uganda	–	–	–	HI	Febrile patients	5	181	–	–
21	Mease^[64]^	Cross-sectional study	Population based	Kenya	731	410	18–25 = 266 26–35 = 286 36–45 = 219 >45 = 270	IgG (ELISA)	No symptom	383	1141	5.7% (65/1141) were DENV+; 2.4% (27/1141) were WNV+; 1.24% (14/1141) were YFV+; 0.72% (8/1141) were RVFV+.	–
22	Mohammed F^[65]^	Cross-sectional study	Hospital based	Khartoum state, Sudan.	48 (1)	42 (1)	5–14 = 6 15–29 = 31 30–44 = 24 45–59 = 19 >60 = 10	IgG/IgM (ELISA)	Febrile symptoms	2	90	2 (2.2%) were IgG+; 0 (0%) for IgM.	–
23	Mohammed, H^[66]^	Cross-sectional study	Hospital based	Singa City Sinnar State Sudan	–	–	21–30 = 46 31–40 = 37 41–50 = 07	IgG/IgM (ELISA)	Healthy blood donor	64	90	47 were IgG+; 05 were IgM + and 12 were IgG and IgM+.	–
24	Mwongula^[67]^	Cross-sectional study	Hospital based	Alupe District, Busia Kenya	171 (–)	213 (–)	Median = 4.5 (2.5–8 IQR) 1–12 yr in range	ELISA	Febrile children	36	384	11.5% (44/384) were PRNT + for CHIKV.	–
25	Ochieng^[68]^	Survey	Population based	Kenya	430 (1)	661 (9)	15–29 = 529 30–49 = 395 50–64 = 167	IgG (ELISA)	No symptom	10	909	5.4% (57/1057) were IgG + for RVFV; 13% (143/1091) were IgG + for DENV.	–
26	Omer^[69]^	Survey	Population based	Gezira State, Sudan	–	–		HI		27	109	0.9% (1/109) were also HI + for SNIV, 8.2% (9/109) were VNT + to CHIKV.	–
27	Rodhain^[70]^	Survey	Population based	Tokora, Namalu and Karamoja, northern Uganda	65	67	Range (20–40)	HI	Healthy individuals	62	132	2.2% (3/132) were HI + for YFV and DENV.	–
28	Salah^[71]^	Survey	Population and hospital based	Djibouti	–	–	–	IFA	50 were healthy soldiers, 69 health inhabitant and 41 sick peoples admitted with acute febrile illness	1	119	The single IFT + subject was a native of Ethiopia and cross-reacted with SIN and other alphaviruses.	–
29	Salim^[72]^	Serological survey	Hospital based	Sennar district, Sudan	51	11	0–10 = 4 11–20 = 10 21–30 = 12 31–40 = 24 >40 = 12	NT	No symptom	8	62	23% (11/48) were also VNT + for ONNV. 6.5% (4/62) for YFV + and 47 (8/17) for WNV+.	–
30	Sergon^[33]^	Cross-sectional study	Population based	Lemu island, Kenya	95 (73)	193 (142)	Mean age = 32	IgG/IgM (ELISA)	–	215	288	No other test were performed	–
31	Surtees^[73]^	Survey	Population based(school)	The Kano Plain, Nyanza Province, Kenya	–	–	Range (4–12)	HI	–	224	624	No other test were performed	Rice irrigation increase mosquito breeding in the area.
32	Sutherland^[74]^	Retrospective study	Population based	Kenya	–	–	15–46 = 419 3–15 = 122	IFA	No symptom	201	663	28.6% (190/663) were IgG + for YFV; 42.7% (283/663) were IgG + for DENV1; 44.8% (297/663) were IgG + for DENV2; 37/1% (246/663) were IgG + for DENV3; 30.3% (201/663) were IgG + for DENV4. All IFA positive CHIKV were PRNT +.	–
33	Tigio^[75]^	Cross-sectional study	Hospital based	Lake Baringo, Lake Naivasha, and Tana, Kenya	155	224	Mean age = 24.4	IgG (ELISA)	Febrile illness symptoms	10	379	Of the 10 ELISA+, 1 (10%) neutralized with CHIKV, 5 (50%) with SFV, 3 (30%) with ONNV, and one (10%) with SINDV. Showed cross-reactivity with SFV, ONNV and SINDV.	–
34	Woodruff^[76]^	Survey	Hospital based	Juba, Sudan	–	–	0–20 = 47 21–40 = 58 41–50 = 15 >50 = 10	HI	All patients had at least 1 episode of non- malarial fever with in previous 6 month.	30	130	3.1% (4/130) were HI + for SNIV; 2.3% (3/130) were HI + for SFV; no observed cross-reaction between CHIKV, SNIV or SFV; 1 observed cross-reaction between SNIV and SFV.	–

(–) = No information, and (+) = positive case, AOR = adjusted odds ratio, CHIKV = chikungunya virus, DENV = dengue virus, ELISA = enzyme-linked immune sorbent assay, HI = hemagglutination inhibition, IFA = immunofluorescence assay, IgG = immunoglobulin G, IgM = immunoglobulin M, NT = neutralization test, ONNV = O’nyong-nyong virus, PRNT = plaque reduction neutralization tests, RT-PCR = reverse transcriptase polymerase chain reaction, RVF = Rift Valley fever, SFV = Semliki Forest virus, SINV = Sindbis virus, WNV = West Nile virus, YFV = Yellow Fever virus, ZIKV = Zika virus.

### 3.2. Risk of bias in studies

Overall, the risk of bias was low in 20%, moderate in 65.7%, and high in 14.3% of the studies. The risk of bias assessments are summarized in Table S2, Supplemental Digital Content, https://links.lww.com/MD/R324.

### 3.3. Pooled seroprevalence of CHIKV

**Figure 2.**
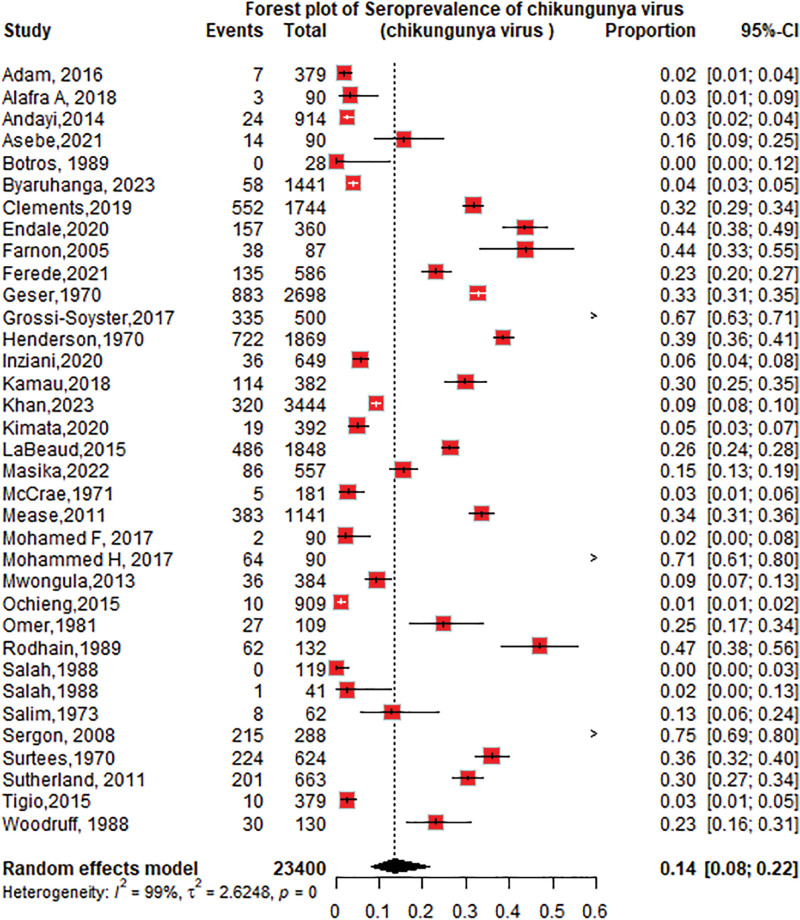
Forest plot of the overall pooled seroprevalence of chikungunya virus in the Horn of Africa.

The overall seroprevalence of CHIKV identified in the 34 studies was 14% (95% CI: 9–23; *I*^2^ = 99%), participants = 23,400 (Fig. 2).

### 3.4. Subgroup analysis

#### 3.4.1. The seroprevalence of CHIKV among countries

**Figure 3.**
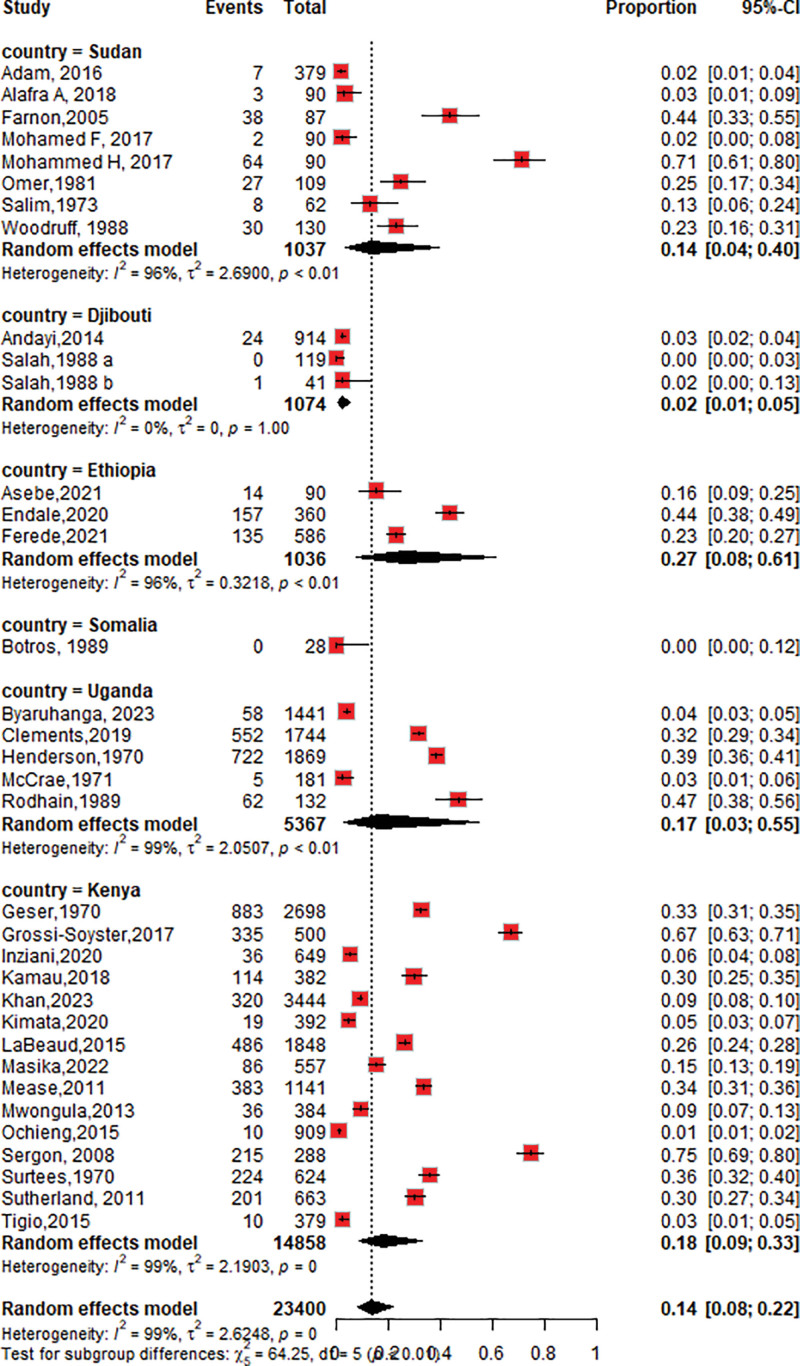
Forest plot of the subgroup analysis of chikungunya virus seroprevalence by country.

The seroprevalence of CHIKV in Ethiopia was 27% (95% CI: 8–61; *I*^2^ = 96%), followed by Kenya at 18% (95% CI: 9–33; *I*^2^ = 99%), Uganda at 17% (95% CI: 3–55; *I*^2^ = 99%), and Sudan at 14% (95% CI: 4–40; *I*^2^ = 96%), as shown in Figure 3.

#### 3.4.2. The seroprevalence of CHIKV among the study population

**Figure 4.**
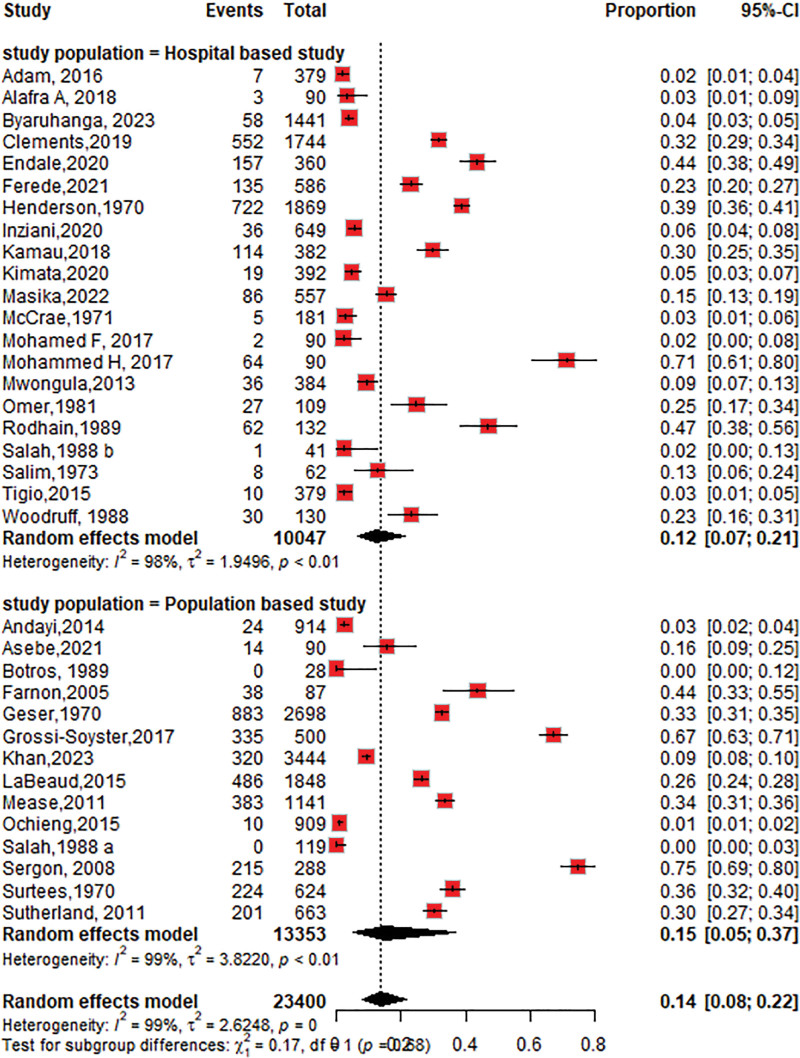
Forest plot of the subgroup analysis of chikungunya virus seroprevalence by study setting (population based vs hospital-based).

The seroprevalence was higher in study conducted in population settings 15% (95% CI: 5–37; *I*^2 =^ 99%) than in those conducted in hospital settings 12% (95% CI: 7–21; *I*^2^ = 98%), as shown in Figure 4.

#### 3.4.3. The seroprevalence of CHIKV among age groups

**Figure 5.**
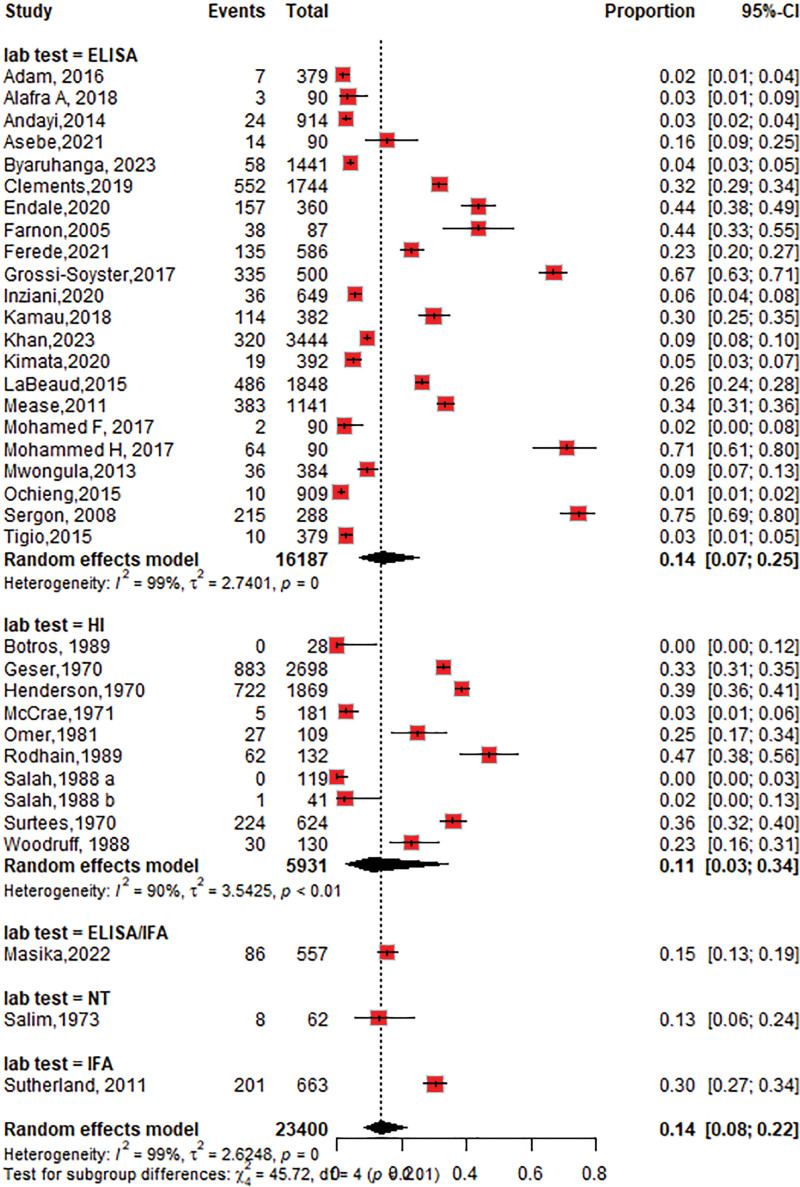
Forest plot of the subgroup analysis of chikungunya virus seroprevalence by age group.

The seroprevalence of CHIKV was higher among adults 40% (95% CI: 13–74; *I*^2^ = 96%) than other age groups, as shown in Figure 5.

#### 3.4.4. The seroprevalence of CHIKV based on diagnostic tests

**Figure 6.**
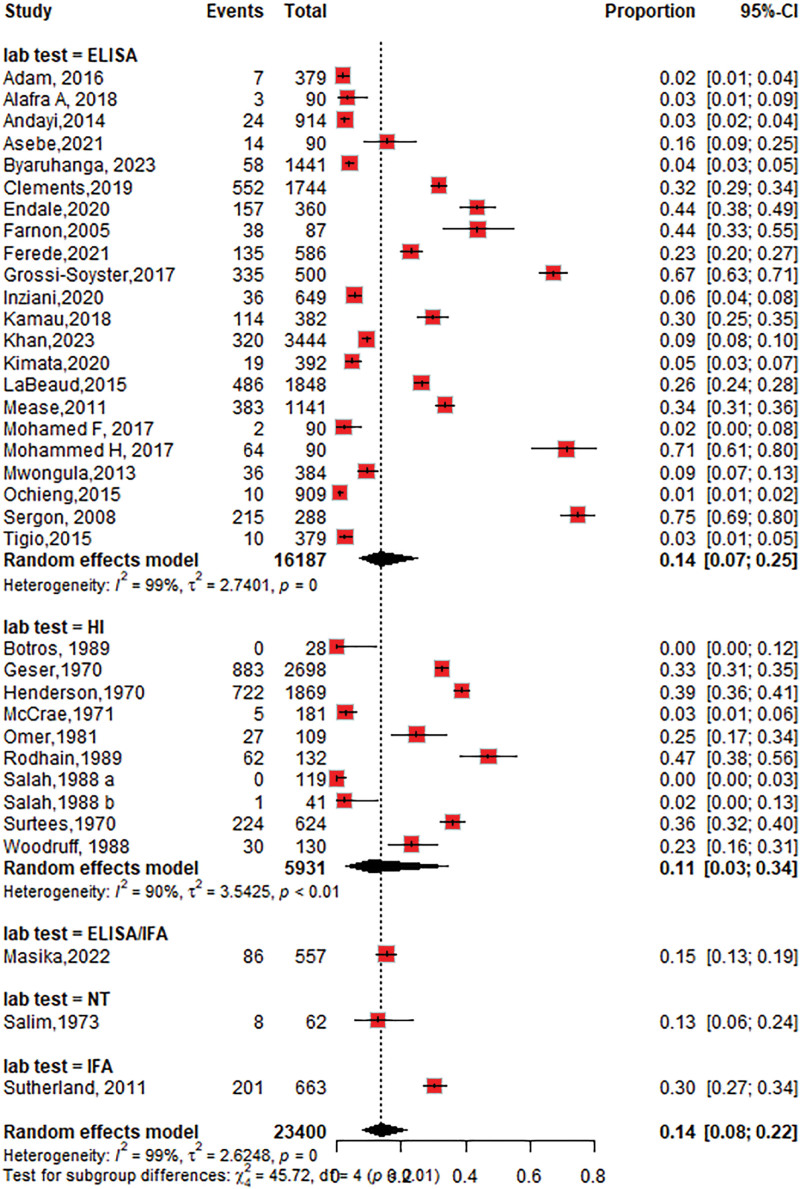
Forest plot of the subgroup analysis of chikungunya virus seroprevalence by diagnostic test category (ELISA vs hemagglutination inhibition). ELISA = enzyme-linked immune sorbent assay.

**Figure 7.**
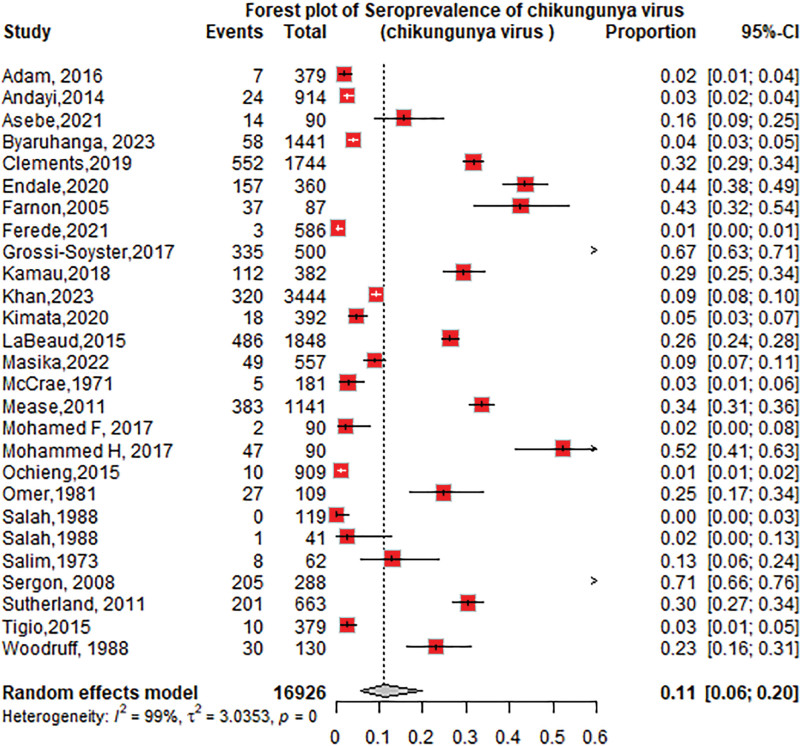
Forest plot of the subgroup analysis of chikungunya virus seroprevalence by IgG ELISA. ELISA = enzyme-linked immune sorbent assay, IgG = immunoglobulin G.

**Figure 8.**
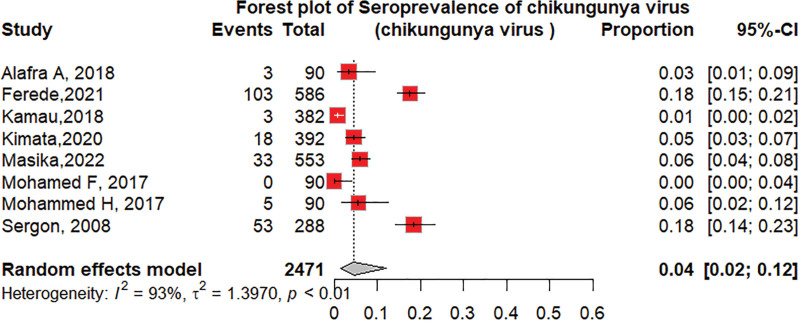
Forest plot of the subgroup analysis of chikungunya virus seroprevalence by IgM ELISA. ELISA = enzyme-linked immune sorbent assay, IgM = immunoglobulin M.

**Figure 9.**
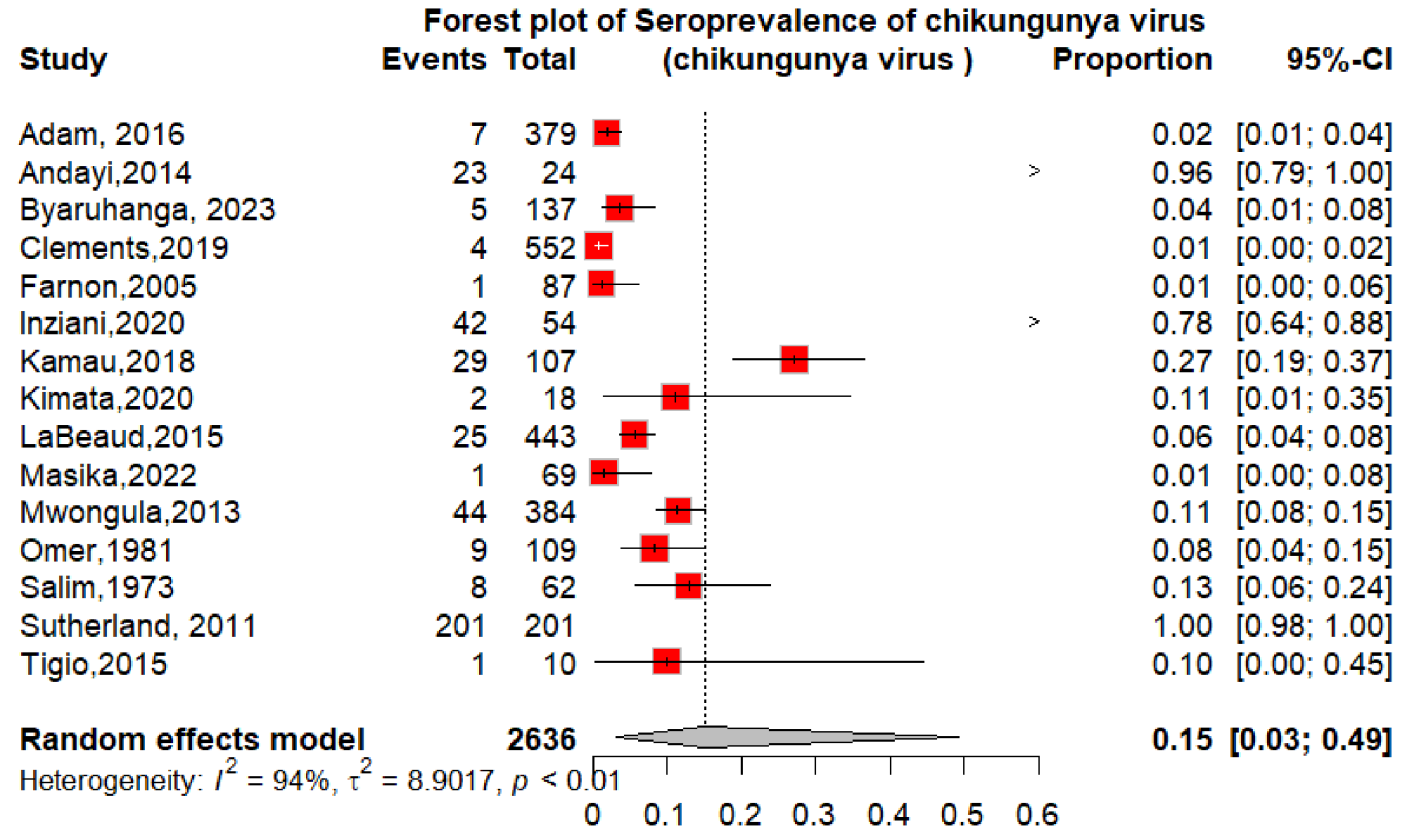
Forest plot of the subgroup analysis of chikungunya virus seroprevalence by plaque reduction neutralization test (PRNT).

The seroprevalence of CHIKV was higher in studies that used ELISA 14% (95% CI: 7–25; *I*^2^ = 99%), followed by the HI test 11% (95% CI: 3–34; *I*^2^ = 90%) as shown in Figure 6. Among the ELISA tests performed, the seroprevalence of chikungunya using IgG was 11% (95% CI: 6–20; *I*^2^ = 99%), followed by IgM at 4% (95% CI: 2–12; *I*^2^ = 93%), as shown in Figures 7 and 8. The seroprevalence of chikungunya in the Horn of Africa among those studies that used the gold standard confirmatory test, PRNT, was 15% (95% CI: 3–49%; *I*^2^ = 94%), as shown in Figure 9. Out of which, 24% of the PRNTs cross-reacted with ONNV only, and 36% of the PRNTs cross-reacted with both CHIKV and ONNV.

#### 3.4.5. The seroprevalence of CHIKV among inapparent infections

**Figure 10.**
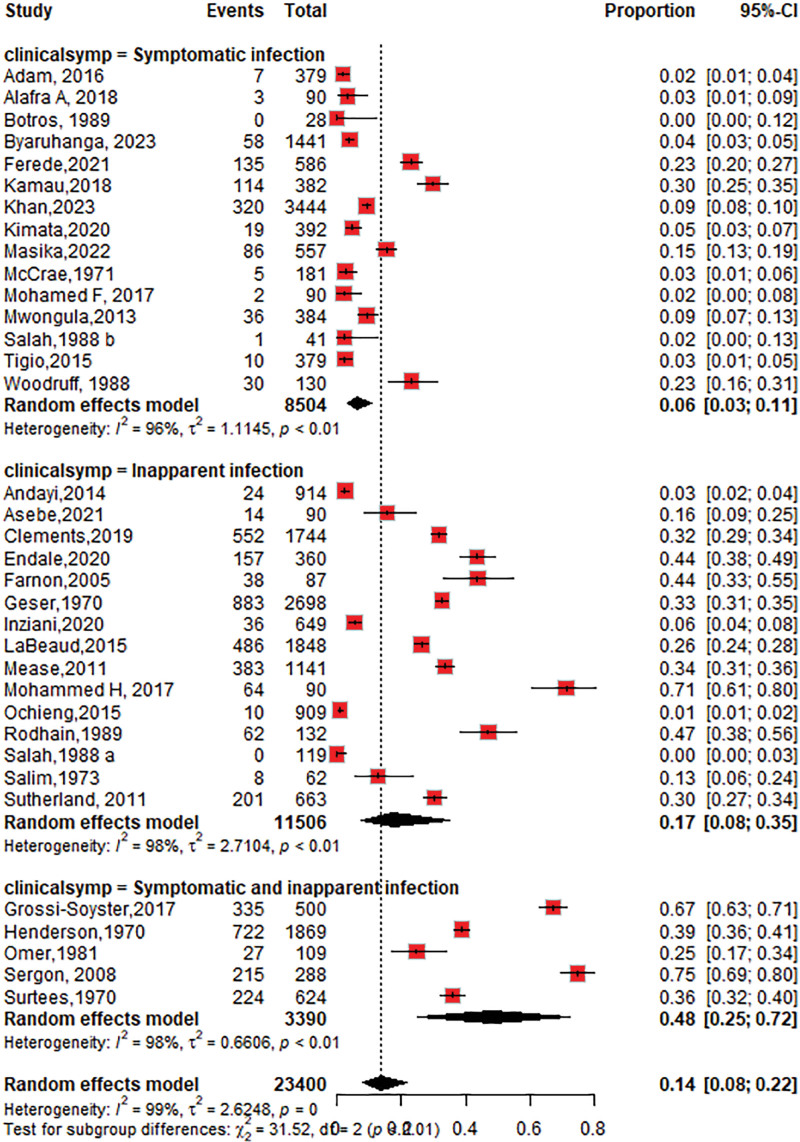
Forest plot of the subgroup analysis of chikungunya virus seroprevalence by clinical manifestation (inapparent vs symptomatic infection).

The seroprevalence of CHIKV among inapparent infections 17% (95% CI: 8–35; *I*^2^ = 98%) was higher than that among symptomatic infection 6% (95% CI: 3–11; *I*^2^ = 96%), as shown in Figure 10.

#### 3.4.6. The trend of CHIKV seroprevalence over a period of time

**Figure 11.**
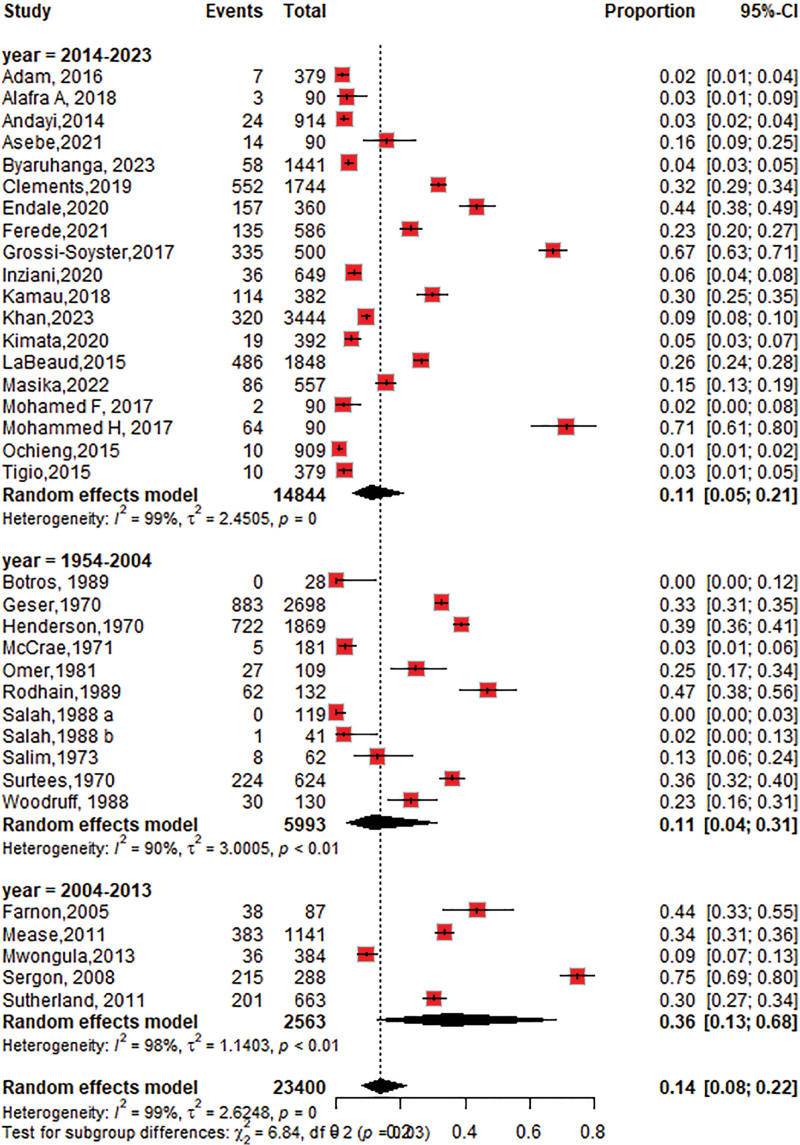
Forest plot of the subgroup analysis of chikungunya virus seroprevalence over time (1954–2004, 2004–2013, 2014–2023).

The seroprevalence of CHIKV was 11% (95% CI: 4–31; *I*^2^ = 90%), 36% (95% CI: 13–68; *I*^2^ = 98%) and 11% (95% CI: 5–21; *I*^2^ = 99%) between 1954 and 2004, 2004 and 2013, and 2014 and 2023, respectively, as shown in Figure 11.

### 3.5. Meta-regression analysis

To better explain the heterogeneity of the chikungunya seroprevalence in the Horn of Africa, a meta-regression analysis that considered several factors at the same time was performed. The meta-regression results revealed that the seroprevalence of CHIKV was highest in Ethiopia 2.8 (95% CI: 0.82–4.8; *P* value: .008), followed by Kenya 2.52 (95% CI: 0.83–4.21; *P* value: .0056) and Sudan 1.92 (95% CI: 0.07–3.78), *P* value = .042. The chikungunya seroprevalence in children was −2.46 (95% CI: −4.43 to −0.49), *P* value = .017 and that in all age groups was −1.68 (95% CI −3.12 to −0.25), *P* value = .023, which was significantly lower than that in adults. The chikungunya seroprevalence rate between 2004 and 2013 was 1.35 (95% CI: 0.18–2.53), and the *P* value was .025, which was higher than that in other periods, as shown in Table 2.

**Table 2:** Meta-regression by country, age group, study setting, laboratory test, time trend in year, and clinical symptom.

Covariates		Coefficient (CI, 95%)	Std. err	*P* value
Intercept		−2.3 (−4.51 to −0.16)	1.0379	.036
Country	Ethiopia***	2.8 (0.82 to 4.8)	0.95	.008
	Kenya***	2.52 (0.83 to 4.21)	0.80	.0056
	Sudan**	1.92 (0.07 to 3.78)	0.88	.042
	Uganda*	1.46 (−0.57 to 3.5)	0.97	.148
	Somalia***	−18.23 (−38343.29 to 38306.83)	18310.85	.99
	Djibouti (reference)			
Age	All age	−1.68 (−3.12 to −0.25)	0.68	.023
	Children	−2.46 (−4.43 to −0.49)	0.94	.017
	Adult (reference)			
Study setting	Hospital-based study (reference)			
	Population based study	−0.37 (−1.47 to 0.73)	0.52	.49
Laboratory test	ELISA & IFA	0.53 (−1.65 to 2.71)	1.04	.61
	HI*	0.40 (−0.60 to 1.40)	0.48	.41
	IFA	−0.32 (−2.55 to 1.90)	1.06	.76
	NT	0.14 (−2.28 to 2.56)	1.15	.91
	ELISA(reference)			
Time trend in years	2004–2013**	1.35 (0.18 to 2.53)	0.56	.025
	2014–2023	−0.10 (−1.33 to 1.13)	0.6055	.86
	1954–2004 (reference)			
Clinical symptom	Symptomatic	−0.74 (−1.85 to 0.37)	0.53	.18
	Inapparent infection (reference)			
Wld (df = 19)		477.063		<.0001

ELISA = enzyme-linked immune sorbent assay, HI = hemagglutination inhibition, IFA = immunofluorescence assay, NT = neutralization test, Std. err = standard error.

* *P* value: .05.

** *P* value: .01.

*** *P* value: .001.

### 3.6. Publication test

**Figure 12.**
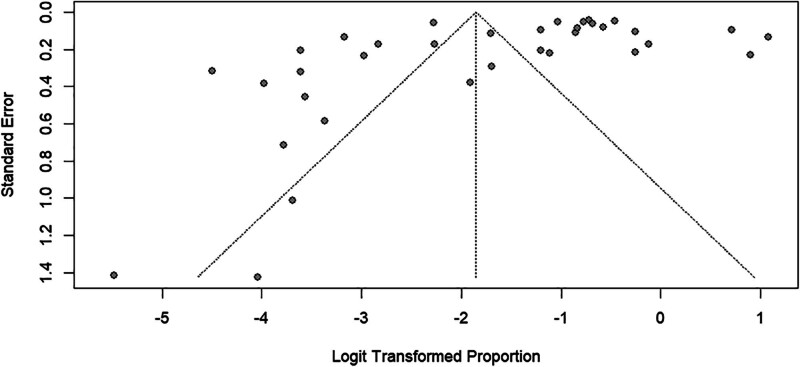
Funnel plot for the assessment of potential publication bias in the included studies.

In the funnel plot, 2 diagonal lines represent 95% confidence limits (effect ± 1.96 SE) around the summary effect for each standard error on the vertical axis. The result showed symmetry around the effect, indicating no publication bias (Fig. 12).

### 3.7. Egger regression test

Egger test showed the possibility of publication bias, with a *P* value of .036. It is lower than .05.

## 4. Discussion

This systematic review and meta-analysis, which included 34 published studies, assessed the seroprevalence of CHIKV in the Horn of Africa. Our review highlights a considerable level of coinfection between CHIKV and other arboviruses, as well as acute febrile illnesses such as malaria. In total, 38% of the isolates were infected with yellow fever virus, 32% with dengue fever, 17.6% with West Nile virus, and 11.7% with Zika and Rift Valley virus. Malaria coinfection occurred in 1 study. Coinfection in the region shows the burden of arboviruses. The vector *A aegypti* may transmit more than arboviruses simultaneously. Even though there is limited clinical data on the implications of co-exposure and co-transmission on the epidemiology, pathogenesis, and evolution of these agents, it affect the vector competence and prevention measure needed to be taken.^[77,78]^

The pooled CHIKV seroprevalence across studies was 14%, indicating a notable disease burden in the Horn of Africa. When stratified by study type, the seroprevalence among community-based populations 15% (95% CI: 5–37; *I*^2^ = 99%) was slightly higher than that among hospital-based studies of 12% (95% CI: 7–22; *I*^2^ = 98%). This result was lower than the seroprevalence reported in the population setting in the World Health Organization Regions (African, Americas, Eastern Mediterranean, European, Southeast Asian, and Western Pacific) of 24% (95% CI: 19–29),^[79]^ but the subgroup analysis revealed that the chikungunya seroprevalence in the African region was 31% (95% CI: 21–41), which is higher than that in our study. According to a systematic review and meta-analysis of studies published from 2000 to 2019, the worldwide pooled seroprevalence of chikungunya was 25% (95% CI: 22–29), while in the African region, it was 41% (95% CI: 29–54),^[80]^ which is higher than what we found in this review. This may be due to the low circulation of the CHIKV in the 1900s and the heterogeneity in population herd immunity in our studies.

Considerable variability in seroprevalence across individual studies was also observed, likely due to differences in sample size, methodology, and study setting. The highest seroprevalence was reported in Kenya (74.6%),^[33]^ followed by Uganda (47%),^[70]^ Ethiopia (44%),^[51]^ and Sudan (44%),^[52]^ while the lowest rate (0%) was reported in Somalia.^[48]^

Subgroup analysis was performed in our review based on country of residence to determine the variation in seroprevalence across the groups and to investigate heterogeneity. The seroprevalence of chikungunya was highest in Ethiopia 27% (95% CI: 8–61), followed by Kenya 18% (95% CI: 9–33), Uganda 17% (95% CI: 3–55), and Sudan 14% (95% CI: 4–39). The variation in the chikungunya seroprevalence among countries could be because of differences in the study setting, and the small studies included in the meta-analysis may exaggerate the result. In addition, the meta-regression results revealed that the relative risk of CHIKV seroprevalence in Ethiopia RR 2.8 and Kenya RR 2.5 was high. It is essential to carefully interpret and understand these differences. The results show that the risk of vector transmission across borders is very high, and there is a likelihood of the disease being introduced into countries through travel or migration and the risk of an outbreak in the region.

The seroprevalence of chikungunya among different age groups showed a higher seroprevalence in adults (40%) than in all age groups (12%) and children (10%). The high chikungunya seroprevalence in adults may be due to behavior, such as outdoor activities and travel to endemic areas.^[81,82]^

Regarding diagnostic methods, CHIKV serology detected by ELISA and/or IF in endemic areas may not be specific because of possible cross-reactivity with other viruses (i.e., ONNV); thus, confirmation with PRNTs is needed to distinguish CHIKV infection from other arboviral infections. Our study showed that the chikungunya seroprevalence among samples tested by ELISA was 14%, followed by that among HI 11%. PRNT showed a similar seroprevalence of 15% over all tests. In addition to the percentage of CHIKV-positive PRNTs, 24% of the PRNTs cross-reacted with ONNV only, and 36% of the PRNTs cross-reacted with both CHIKV and ONNV. The results showed that serologic tests, such as ELISA, IFA, and HI tests, overestimated the seroprevalence of CHIKV, and confirmatory tests, such as PRNT, and molecular tests, such as RT-PCR, are needed to accurately determine the seroprevalence.

The review showed that inapparent or asymptomatic infections play a significant role in chikungunya seroprevalence studies and have important implications for disease transmission, public health planning, and understanding the true burden of CHIKV within a population. Our study showed that the chikungunya seroprevalence in 17% of patients with inapparent infections was higher than that in 6% of patients with symptomatic infections. A meta-analysis of worldwide chikungunya seroprevalence revealed that the incidence of inapparent infection was 40%, whereas it was 26% in Africa.^[80]^ Because these patients do not seek medical attention or are not tested, they are often missed by surveillance systems, leading to an underestimation of the chikungunya seroprevalence. Asymptomatic individuals may also be less likely to adhere to preventive measures, such as vector control strategies, further facilitating virus transmission and posing challenges for public health planning and response efforts.

Since its discovery in 1954, CHIKV seroprevalence and transmission patterns have evolved considerably due to globalization, urbanization, climate change, and vector control efforts.^[83]^ The trends were analyzed for 3 periods: 1954 to 2004, 2004 to 2013, and 2014 to 2023. Our temporal trend analysis revealed a 35% increase in seroprevalence during 2004 to 2013, coinciding with the reemergence of CHIKV in Kenya (2004) and subsequent large-scale outbreaks across new regions. These findings highlight how ecological and demographic changes have shaped the virus’s reemergence and spread.

This study represents the 1st comprehensive meta-analysis of CHIKV seroprevalence across the Horn of Africa, integrating a large dataset and extensive subgroup analyses. However, some limitations exist: data were unavailable for certain countries, and most included studies were conducted during outbreak periods, which may have biased prevalence estimates. Additionally, high heterogeneity across studies may reflect differences in study design, diagnostic methods, and reporting quality.

## 5. Conclusion

Our review provides compelling evidence of CHIKV circulation in the Horn of Africa, revealing diverse seroprevalence rates across different countries, age groups, laboratory tests, clinical manifestations, and periods. The high rate of arboviral coinfection underscores the region’s heavy arbovirus burden and the complex challenges facing vector control programs. Confirmatory tests such as PRNT should be performed for all serologic tests for better diagnosis. This study contributes to a better understanding of the actual burden of chikungunya infections, laying the groundwork for informed decisions in future prevention measures. Collaboration among countries contributes to success in global health security. Cross-border surveillance of *Aedes* mosquito species and asymptomatic cases should be implemented to prevent outbreaks and reduce the risk of infection.

## Acknowledgments

We would like to express our gratitude to the Center for Innovative Drug Development and Therapeutic Trials for Africa (CDT-Africa), College of Health Sciences, Addis Ababa University, for supporting this study.

## Author contributions

**Conceptualization:** Yishak Abraham.

**Data curation:** Yishak Abraham, Kibrom Abraham, Monica S. Kahabuka, Mesoud A. Bushara, Firehiwot Ayenadis.

**Formal analysis:** Yishak Abraham, Dawit Getachew Assefa, Monica S. Kahabuka, Esther Nthenya Muthoka, Seke G.Y. Muzazu.

**Methodology:** Yishak Abraham.

**Software:** Yishak Abraham, Dawit Getachew Assefa.

**Supervision:** Dawit Getachew Assefa, Seke G.Y. Muzazu, Mesoud A. Bushara, Tsegahun Manyazewal.

**Validation:** Yishak Abraham, Tsegahun Manyazewal.

**Visualization:** Yishak Abraham.

**Writing – original draft:** Yishak Abraham.

**Writing – review & editing:** Yishak Abraham, Dawit Getachew Assefa, Kibrom Abraham, Monica S. Kahabuka, Esther Nthenya Muthoka, Seke G.Y. Muzazu, Firehiwot Ayenadis, Tsegahun Manyazewal.

## Supplementary Material


