# Supplementary material for: Chikungunya seroprevalence in the Horn of Africa: A systematic review and meta-analysis
Source: Medicine (Baltimore). 2026 Feb 6;105(6):e47564. doi: 10.1097/MD.0000000000047564 (PMC12885733; doi:10.1097/MD.0000000000047564)
Supplement: Supplementary file 1 [file medi-105-e47564-s001.docx]

**Supplementary material 1: Shows diagnostic laboratory test performed in the studies.**

| **Author’s name , Date** | **Laboratory test** | | | | | | | **Index test (Commercial/ In-house)** |
| --- | --- | --- | --- | --- | --- | --- | --- | --- |
|  | **ELISA** | | | **PRNT** | **IFA** | **HI** | **RT-PCR** |  |
|  | **Ig G** | **Ig M** | **IgG/**  **IgM** |  |  |  |  |  |
| **Adam, 2016^(45)^** | 7/379 |  |  | 7/379 | 7/379 |  |  | Commercial anti-CHIKV |
| **Alafra A, 2018^(46)^** |  | 3/90 |  |  |  |  | 6/90 | Commercial anti-CHIKV |
| **Andayi, 2014^(44)^** | 24/914 |  |  | 23/24 |  |  |  | In-house kits |
| **Asebe, 2021^(47)^** | 14/90 |  |  |  |  |  |  | Commercial anti-CHIKV |
| **Botros, 1989^(48)^** |  |  |  |  | 0/28 | 0/28 |  | Commercial anti-CHIKV |
| **Byaruhanga, 2023^(49)^** | 58/1441 |  |  | 5/137 |  |  |  |  |
| **Clements, 2019^(50)^** | 552/1744 |  |  | 4/552 |  |  |  |  |
| **Endale, 2020^(51)^** | 157/  360 |  |  |  |  |  |  |  |
| **Farnon, 2005^(52)^** | 37/87 |  |  | 1/87 |  |  |  |  |
| **Ferede, 2021^(53)^** | 3/586 | 103/586 | 28/586 |  |  |  |  |  |
| **Geser, 1970^(54)^** |  |  |  |  |  | 883/ 2,698 |  |  |
| **Grossi-Soyster, 2017^(55)^** | 335/ 499 |  |  |  |  |  |  |  |
| **Henderson, 1970^(56)^** |  |  |  |  |  | 722/1,869 |  |  |
| **Inziani, 2020^(57)^** |  |  | 36/649 | 42/54 |  |  |  | In-house kit |
| **Kamau, 2018^(58)^** | 112/382 | 3/382 | 1/382 | 29/107 |  |  |  | In-house kit |
| **Khan, 2023^(59)^** | 320/ 3,444 |  |  |  |  |  |  |  |
| **Kimata, 2020^(60)^** |  | 18/392 | 1/392 | 2/18 |  |  |  | Commercial anti-CHIKV |
| **LaBeaud, 2015^(61)^** | 486/  1848 |  |  | 25/443 |  |  |  |  |
| **Masika, 2022^(62)^** |  | 33/553 | 4 | 1/69 | 49/557  (**IgG**) |  |  | In-house IFA and a commercial ELISA |
| **McCrae, 1971^(63)^** |  |  |  |  | 5/181 |  |  |  |
| **Mease, 2011^(64)^** | 383/ 1,141 |  |  |  |  |  |  |  |
| **Mohammed F, 2017^(65)^** | 2/90 | 0/90 |  |  |  |  |  |  |
| **Mohammed, H 2017^(66)^** | 47/90 | 5/90 | 12/90 |  |  |  |  |  |
| **Mwongula, 2013^(67)^** |  |  | 36/384 | 44/384 |  |  |  |  |
| **Ochieng, 2015^(68)^** | 10/909 |  |  |  |  |  |  | Commercial anti-CHIKV |
| **Omer, 1981^(69)^** |  |  |  | 9/109 | 27/109  (**IgG**) |  |  |  |
| **Rodhain, 1989^(70)^** |  |  |  |  | 62/132 |  |  |  |
| **Salah, 1988^(71)^** |  |  |  |  |  | 1/119  (**IgG**) |  |  |
| **Salim, 1973^(72)^** |  |  |  | 8/62  (**IgG**) |  |  |  |  |
| **Sergon, 2008^(33)^** | 206/288 | 53/288 | 215/288 |  |  |  |  |  |
| **Surtees, 1970^(74)^** |  |  |  |  | 224/624 |  |  |  |
| **Sutherland, 2011^(73)^** |  |  |  | 201/201 |  | 201/  663 |  |  |
| **Tigio, 2015^(75)^** | 10/379 |  |  | 1/10 |  |  |  |  |
| **Woodruff, 1988^(76)^** |  |  |  |  | 30/130  (**IgG**) |  |  |  |

**ELISA**: Enzyme-Linked Immune Sorbent Assay, **HI**: hemagglutination inhibition, **IFA**: immunofluorescence assay, **IgG**: immunoglobulin G, **IgM**: immunoglobulin M, and **RT-PCR**: reverse transcriptase polymerase chain reaction

**Supplementary material 2: The Agency for Healthcare Research and Quality (AHRQ) with an 11-item checklist for cross-sectional study.**

| **No** | **Items** | **Articles** | | | | | | | | | | | | | | | | | | | | | | | | | | | | | | | | | | |
| --- | --- | --- | --- | --- | --- | --- | --- | --- | --- | --- | --- | --- | --- | --- | --- | --- | --- | --- | --- | --- | --- | --- | --- | --- | --- | --- | --- | --- | --- | --- | --- | --- | --- | --- | --- | --- |
|  |  | **Adam, 2016** | **Alafra A, 2018** | **Andayi,2014** | **Asebe,2021** | **Botros, 1989** | **Bower,2021** | **Byaruhanga, 2023** | **Clements,2019** | **Endale,2020** | **Farnon,2005** | **Ferede,2021** | **Geser,1970** | **Grossi- Soyster**  **,2017** | **Henderson,1970** | **Inziani,2020** | **Kamau,2018** | **Khan,2023** | **Kimata,2020** | **LaBeaud,2015** | **Masika,2022** | **McCrae,1971** | **Mease,2011** | **Mohamed F, 2017** | **Mohamed H, 2017** | **Mwongula,2013** | **Ochieng,2015** | **Omer,1981** | **Rodhain,1989** | **Salah,1988** | **Salim,1973** | **Sang,2011** | **Sergon, 2008** | **Surtees,1970** | **Tigio,2015** | **Woodruff, 1988** |
| **1** | **Define the source of information.** | **+** | **+** | **+** | **+** | **+** | **+** | **+** | **+** | **+** | **+** | **+** | **+** | **+** | **+** | **+** | **+** | **+** | **+** | **+** | **+** | **+** | **+** | **+** | **+** | **+** | **+** | **+** | **+** | **+** | **+** | **+** | **+** | **+** | **+** | **+** |
| **2** | **List inclusion and exclusion criteria for exposed and unexposed subjects (cases and controls) or refer to previous publications** | **-** | **-** | **+** | **+** | **-** | **+** | **+** | **-** | **+** | **+** | **+** | **+** | **+** | **-** | **+** | **+** | **+** | **-** | **+** | **-** | **-** | **+** | **+** | **+** | **+** | **+** | **+** | **+** | **-** | **-** | **-** | **+** | **-** | **+** | **+** |
| **3** | **Indicate time period used for identifying patients** | **+** | **+** | **+** | **+** | **+** | **+** | **+** | **+** | **+** | **+** | **+** | **+** | **+** | **+** | **+** | **+** | **+** | **+** | **+** | **+** | **+** | **+** | **+** | **+** | **+** | **+** | **+** | **+** | **+** | **+** | **+** | **+** | **+** | **+** | **+** |
| **4** | **Indicate whether or not subjects were consecutive if not population-based** | **+** | **+** | **+** | **+** | **+** | **+** | **+** | **+** | **+** | **+** | **+** | **+** | **+** | **+** | **+** | **+** | **+** | **+** | **+** | **+** | **+** | **+** | **+** | **+** | **+** | **+** | **+** | **+** | **+** | **+** | **+** | **+** | **+** | **+** | **+** |
| **5** | **Indicate if evaluators of subjective components of study were masked to other aspects of the status of the participants** | NA | NA | NA | NA | NA | NA | NA | NA | NA | NA | NA | NA | NA | NA | NA | NA | NA | NA | NA | NA | NA | NA | **-** | NA | NA | NA | NA | NA | NA | NA | NA | NA | NA | NA | NA |
| **6** | **Describe any assessments undertaken for quality assurance purposes** | **+** | **+** | **+** | **-** | **+** | **+** | **+** | **+** | **-** | **+** | **+** | **+** | **-** | **-** | **+** | **+** | **+** | **+** | **+** | **+** | **-** | **+** | **-** | **-** | **+** | **-** | **+** | **-** | **-** | **-** | **+** | **-** | **-** | **+** | **-** |
| **7** | **Explain any patient exclusions from analysis** | **-** | **-** | **-** | **-** | **-** | **-** | **+** | **-** | **-** | **-** | **-** | **-** | **-** | **-** | **-** | **-** | **-** | **-** | **+** | **+** | **-** | **+** | **-** | **-** | **-** | **-** | **-** | **+** | **-** | **-** | **-** | **-** | **-** | **-** | **-** |
| **8** | **Describe how confounding was assessed and/or controlled.** | **-** | **-** | **+** | **+** | **-** | **+** | **+** | **NA** | **+** | **+** | **+** | **+** | **+** | **-** | **+** | **+** | **+** | **-** | **+** | **+** | **-** | **+** | **-** | **+** | **+** | **?** | **-** | **-** | **-** | **-** | **-** | **-** | **-** | **+** | **+** |
| **9** | **If applicable, explain how missing data were handled in the analysis** | **NA** | **NA** | **+** | **-** | **NA** | **NA** | **NA** | **NA** | **NA** | **-** | **NA** | **NA** | **NA** | **-** | **-** | **-** | **NA** | **-** | **+** | **-** | **-** | **+** | **-** | **-** | **-** | **-** | **-** | **-** | **-** | **-** | **-** | **-** | **-** | **-** | **-** |
| **10** | **Summarize patient response rates and completeness of data collection** | **+** | **+** | **+** | **+** | **+** | **+** | **+** | **+** | **+** | **+** | **+** | **+** | **+** | **+** | **+** | **+** | **+** | **+** | **+** | **+** | **+** | **+** | **+** | **+** | **+** | **+** | **+** | **+** | **+** | **+** | **+** | **+** | **+** | **+** | **+** |
| **11** | **Clarify what follow-up, if any, was expected and the percentage of patients for which incomplete data or follow-up was obtained** | **NA** | **NA** | **NA** | **NA** | **NA** | **+** | **NA** | **NA** | **-** | **+** | **-** | **-** | **-** | **-** | **-** | **-** | **+** | **-** | **+** | **-** | **-** | **+** | **-** | **-** | **-** | **-** | **-** | **-** | **-** | **-** | **-** | **-** | **-** | **-** | **-** |
| **Total scores** | | 5 | 5 | 8 | 6 | 5 | 8 | 8 | 5 | 5 | 8 | 7 | 7 | 6 | 4 | 7 | 7 | 8 | 5 | 10 | 7 | 4 | 10 | 5 | 6 | 7 | 5 | 6 | 6 | 4 | 4 | 5 | 5 | 4 | 7 | 6 |
| **Quality of bias** | | M | M | L | M | M | L | L | M | M | L | M | M | M | H | M | M | L | M | L | M | H | L | M | M | M | M | M | M | H | H | M | M | H | M | M |

**Yes = +; No = -; Unclear =? ; Not applicable = NA, H=high risk, M=medium risk and L=low risk**
